# Risk Factors of Psychological Responses of Chinese University Students During the COVID-19 Outbreak: Cross-sectional Web-Based Survey Study

**DOI:** 10.2196/29312

**Published:** 2021-07-21

**Authors:** Xudong Zhang, Xin Shi, Yang Wang, Huiquan Jing, Qingqing Zhai, Kunhang Li, Dan Zhao, Shiyu Zhong, Yuequn Song, Feng Zhang, Yijun Bao

**Affiliations:** 1 Department of Neurosurgery The Fourth Hospital of China Medical University Shenyang China; 2 Business School All Saints Campus Manchester Metropolitan University Manchester United Kingdom; 3 Department of Social Medicine School of Public Health China Medical University Shenyang China; 4 School of Public Health Capital Medical University Beijing China; 5 School of Management Shanghai University Shanghai China; 6 Department of Neurosurgery The First Hospital of China Medical University Shenyang China

**Keywords:** university students, depressive symptoms, anxiety symptoms, mental health status, COVID-19, pandemic, mental health, anxiety, psychological health

## Abstract

**Background:**

COVID-19 is a highly contagious and highly pathogenic disease caused by a novel coronavirus, SARS-CoV-2, and it has become a pandemic. As a vulnerable population, university students are at high risk during the epidemic, as they have high mobility and often overlook the severity of the disease because they receive incomplete information about the epidemic. In addition to the risk of death from infection, the epidemic has placed substantial psychological pressure on the public. In this respect, university students are more prone to psychological problems induced by the epidemic compared to the general population because for most students, university life is their first time outside the structure of the family, and their mental development is still immature. Internal and external expectations and academic stress lead to excessive pressure on students, and unhealthy lifestyles also deteriorate their mental health. The outbreak of COVID-19 was a significant social event, and it could potentially have a great impact on the life and the mental health of university students. Therefore, it is of importance to investigate university students’ mental health status during the outbreak of COVID-19.

**Objective:**

The principal objective of this study was to investigate the influencing factors of the psychological responses of Chinese university students during the COVID-19 outbreak.

**Methods:**

This study used data from a survey conducted in China between February 21 and 24, 2020, and the data set contains demographic information and psychological measures including the Self-Rating Anxiety Scale, the Self-Rating Depression Scale, and the compulsive behaviors portion of the Yale-Brown Obsessive-Compulsive Scale. A total of 2284 questionnaires were returned, and 2270 of them were valid and were used for analysis. The Mann-Whitney *U* test for two independent samples and binary logistic regression models were used for statistical analysis.

**Results:**

Our study surveyed 563 medical students and 1707 nonmedical students. Among them, 251/2270 students (11.06%) had mental health issues. The results showed that contact history of similar infectious disease (odds ratio [OR] 3.363, *P*=.02), past medical history (OR 3.282, *P*<.001), and compulsive behaviors (OR 3.525, *P*<.001) contributed to the risk of mental health issues. Older students (OR 0.928, *P*=.02), regular daily life during the epidemic outbreak (OR 0.410, *P*<.001), exercise during the epidemic outbreak (OR 0.456, *P*<.001), and concern related to COVID-19 (OR 0.638, *P*=.002) were protective factors for mental health issues.

**Conclusions:**

According to the study results, mental health issues have seriously affected university students, and our results are beneficial for identifying groups of university students who are at risk for possible mental health issues so that universities and families can prevent or intervene in the development of potential mental health issues at the early stage of their development.

## Introduction

A novel coronavirus pneumonia disease, COVID-19, spread very quickly across China in early 2020 [1]. The outbreak was first discovered in late December 2019, when a series of unexplained pneumonia cases were identified that were related to epidemiologically undiscovered seafood market exposure in Wuhan City, Hubei Province, China [2]. According to the official website of the National Health Commission of the People’s Republic of China, on July 31, 2020, a total of 84,337 confirmed cases had accumulated, including 78,989 discharged cases and 4634 deaths; a total of 789,742 close contacts were tracked, and 20,278 close contacts were still under medical observation [3]. The World Health Organization declared the COVID-19 outbreak a public health emergency of international concern on January 30, 2020 [4]. More than 83 million confirmed infection cases across more than 200 countries globally had been reported as of December 31, 2020, including over 1.82 million deaths [5]. The advanced and rapid spread of COVID-19 has brought many complex challenges to the global public health and medical communities.

COVID-19 has brought the risk of death from infection and unbearable psychological pressure to people worldwide [6]. Previous research has shown that the COVID-19 has had a broad psychosocial impact on humans at the individual level, where people may feel fear of illness or death, helplessness, and stigma [7]. During a public health emergency, approximately 10% to 30% of the public are very concerned or quite concerned about the possibility of contracting the disease [8]. As schools and businesses are closed due to the pandemic, individuals’ negative emotions become more complicated [9]. Many studies investigated the psychological impact on uninfected communities during the severe acute respiratory syndrome (SARS) outbreak and found a significant mental illness incidence [10]. Studies also found some risk factors for the deterioration of mental health: being female [11,12], having a medical history, such as prior psychiatric illness, physical illness, or chronic disease [13-15], being unmarried [16-18], having lower income [13,16,19], and experiencing a negative economic impact [20]. People who were more likely to take preventive measures against the infection were older, female, and highly educated; they also had intensive awareness of SARS, a moderate level of anxiety, and a positive contact history [21].

An essential part of the population is university students, who are under heavy study pressure and have unhealthy lifestyles; more importantly, the university stage is a stage of transition to maturity in life development. During university studies, the social knowledge that students have acquired may be insufficient to understand the pandemic due to the limited social activities of students compared with that of working adults [22]. Therefore, the students are not inclined to find ways to release pressure, which can lead to unstable mental states, and the situation can worsen in epidemics such as the COVID-19 pandemic. Besides, the sources of COVID-19 infection may be study places at the university, and populations of hundreds of millions of students are at risk of spreading the virus [23]. Many studies have shown that the outbreak of infectious diseases will have a psychological impact on the general population, including medical staff and university students. A prominent example is the mental sequelae observed during the outbreak of SARS in 2003 [24]. Studies of the SARS outbreak have shown that medical staff experienced acute stress reactions [25,26]. However, medical students also require attention because they are students with fragile mental endurance and medical workers without complete medical training, and their exposure risks are higher than those of other people.

Therefore, the aim of our study is to investigate the mental health of university students and its influencing factors during the COVID-19 pandemic on groups of both medical and nonmedical students. In this study, we conducted a survey of the targeted sample with the aims to explore risk factors that contributed to mental disease during the pandemic and to provide evidence for psychological intervention programs for university students. This research is essential for students’ healthy growth and is an effective response to future work and mental health interventions for students.

## Methods

### Study Population and Sample

The targeted population included medical students from 57 universities in China. In this study, a cross-sectional survey was developed, and anonymous web-based questionnaires were used to investigate students’ mental health status during the COVID-19 epidemic. A snowball sampling strategy was used; the web-based survey was first distributed to medical students, and they were encouraged to pass it on to others. A total of 2284 questionnaires were returned, and 14 of them were excluded because the respondents did not fill in the answers completely or did not meet the criteria of the survey; for example, some respondents were teachers and not students. A total of 2270 valid questionnaires were finalized in the study, including surveys from 563 medical students and 1707 nonmedical students.

### Study Instruments

The questionnaire contained the demographic information and psychological measures, which include the Self-Rating Anxiety Scale (SAS), the Self-Rating Depression Scale (SDS), and the compulsive behaviors part of the Yale-Brown Obsessive-Compulsive Scale (YBOCS).

#### Demographic Information

The questions on demographic information in this study were related to gender, age, whether the respondent is an only child, ethnicity, place of residence, region of residence, whether the respondent has participated in volunteer work, contact history of similar infectious disease, past medical history, regularity of daily life and exercise during the epidemic, and concern about COVID-19. The geographical distribution map of the participants was depicted by ArcGIS software (Esri), and the cutoff points for classification were based on the Jenks classification technique as employed in ArcGIS, version 10.5.

#### The SAS and SDS

The SAS and SDS [27,28] were developed by William WK Zung, a psychiatrist at Duke University. Both the SAS and SDS are Likert scale surveys. They each contain 20 items of self-report examination that measure the level of anxiety symptoms (SAS) or depressive symptoms (SDS). The scores of the 20 items are added in each scale and then converted into standard scores, where higher scores represent more severe anxiety or depression. Based on the Chinese norm, people who score more than 50 on the SAS scale are defined as having anxiety symptoms, and people who score more than 53 on the SDS are treated as having depressive issues. The SAS demonstrated good internal consistency (Cronbach ɑ=.828), and the SDS also showed good internal consistency (Cronbach ɑ=.849).

#### The YBOCS

The YBOCS was developed to remedy problems with existing rating scales by providing a specific measure of the severity of symptoms of obsessive-compulsive disorder (OCD) [29]. This measure ensures that the mental disorder will not be influenced by the type of obsessions or compulsions present. The scale is clinician rated and includes 10 items; a score >6 indicates compulsive behavior. In this study, we used only part of the compulsive behaviors scale of the YBOCS, and it demonstrated good internal consistency (Cronbach ɑ=.810).

### Data Analysis

Data were analyzed using SPSS, version 26.0 (IBM Corporation). According to the data type, the Mann-Whitney *U* test, a type of nonparametric test, was used to explore the significant associations between sample characteristics and mental health issues during the COVID-19 epidemic. Binary logistic regression analysis was conducted for dependent variables (mental health issues) and independent variables (demographic and psychological measures), and we set the level of statistical significance as *P*<.05.

### Ethics Approval and Consent to Participate

This study was conducted in compliance with the Declaration of Helsinki’s ethical principles and its later amendments, and the study was reviewed and approved by the Ethics Committee on Human Experimentation of China Medical University (EC-2020-KS-025). The study procedures followed ethical standards. The participants were informed of the study protocol, and consent was received from all the participants. All participation was voluntary and anonymous. Confidentiality was ensured in processing personal data and maintaining individual records.

## Results

**Figure 1:**
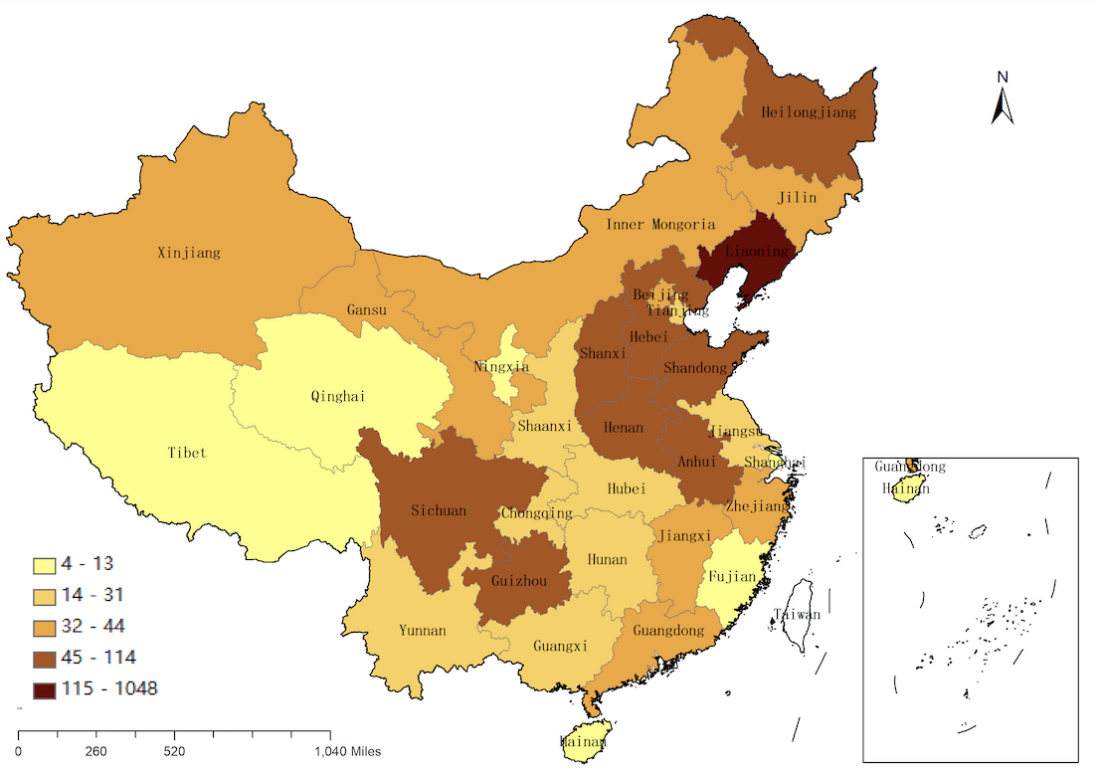
Geographical distribution map of the 2270 study participants.

The characteristics and other demographic information in the valid sample of 2270 respondents are summarized in Table 1. In this study, a new dependent variable (mental health issues) was created to evaluate which independent variables affected the mental health of university students, and the samples with positive mental health issues included students with both positive anxiety symptoms and positive depression symptoms. The results showed that 251 out of 2270 students (11.1%) had mental health issues; among these 251 students, 106 were male (42.2%) and 145 were female (57.8%). Of these 251 students, 214 (85.3%) were between 19 and 24 years of age. The distribution of participants covered the country of China, as shown in Figure 1.

The Mann-Whitney *U* test shows that age, contact history of similar infectious disease, past medical history, compulsive behaviors, the regularity of daily life during the epidemic outbreak, exercise during the epidemic outbreak, and concern about COVID-19 were correlated with mental health issues (all *P*<.05), as shown in Table 2. However, gender, being an only child, ethnicity, place of residence, region, joining in volunteer work, and student type (medical vs nonmedical) had no statistically significant associations with mental health issues (all *P*>.05).

**Table 1:** Distribution of anxiety symptoms, depressive symptoms, and mental health issues among students (N=2270).

Variable			Total, n (%)^a^		Anxiety symptoms, n (%)				Depressive symptoms, n (%)				Mental health issues, n (%)		
				Positive		Negative		Positive		Negative		Positive		Negative	
**Gender**															
	Male	877 (38.6)		47 (5.4)		830 (94.6)		96 (10.9)		781 (89.1)		106 (12.1)		771 (87.9)	
	Female	1393 (61.4)		39 (2.8)		1354 (97.2)		141 (10.1)		1252 (89.9)		145 (10.4)		1248 (89.6)	
**Age (years)**															
	<18	250 (11)		10 (4)		240 (96)		31 (12.4)		219 (87.6)		33 (13.2)		217 (86.8)	
	19-24	1926 (84.8)		73 (3.8)		1853 (96.2)		204 (10.6)		1722 (89.4)		214 (11.1)		1712 (88.9)	
	≥25	94 (4.1)		3 (3.2)		91 (96.8)		2 (2.1)		92 (97.9)		4 (4.3)		90 (95.7)	
**Only child**															
	Yes	1051 (46.3)		43 (4.1)		1008 (95.9)		112 (10.7)		939 (89.3)		120 (11.4)		931 (88.6)	
	No	1219 (53.7)		43 (3.5)		1176 (96.5)		125 (10.3)		1094 (89.7)		131 (10.7)		1088 (89.3)	
**Ethnicity**															
	Han	1936 (85.3)		76 (3.9)		1860 (96.1)		206 (10.6)		1730 (89.4)		215 (11.1)		1721 (88.9)	
	Minority	334 (14.7)		10 (3.0)		324 (97.0)		31 (9.3)		303 (90.7)		36 (10.8)		298 (89.2)	
**Place of residence**															
	Urban	938 (41.3)		34 (3.6)		904 (96.4)		98 (10.4)		840 (89.6)		103 (11.0)		835 (89.0)	
	Rural	1332 (58.7)		52 (3.9)		1280 (96.1)		139 (10.4)		1193 (89.6)		148 (11.1)		1184 (88.9)	
**Region**															
	Hubei Province	26 (1.1)		1 (3.8)		25 (96.2)		2 (7.7)		24 (92.3)		2 (7.7)		24 (92.3)	
	Outside Hubei Province	2244 (99)		85 (3.8)		2159 (96.2)		235 (10.5)		2009 (89.5)		249 (11.1)		1995 (88.9)	
**Joined in volunteer work**															
	Yes	246 (10.8)		9 (3.7)		237 (96.3)		30 (12.2)		216 (87.8)		31 (12.6)		215 (87.4)	
	No	2024 (89.2)		77 (3.8)		1947 (96.2)		207 (10.2)		1817 (89.8)		220 (10.9)		1804 (89.1)	
**Contact history of similar infectious disease**															
	Yes	23 (1)		4 (17.4)		19 (82.6)		8 (34.8)		15 (65.2)		8 (34.8)		15 (65.2)	
	No	2247 (99)		82 (3.6)		2165 (96.4)		229 (10.2)		2018 (89.8)		243 (10.8)		2004 (89.2)	
**Past medical history**															
	Yes	101 (4.4)		12 (11.9)		89 (88.1)		27 (26.7)		74 (73.3)		29 (28.7)		72 (71.3)	
	No	2169 (95.6)		74 (3.4)		2095 (96.6)		210 (9.7)		1959 (90.3)		222 (10.2)		1947 (89.8)	
**Compulsive behaviors**															
	Yes	313 (13.8)		35 (11.2)		278 (88.8)		76 (24.3)		237 (75.7)		81 (25.9)		232 (74.1)	
	No	1957 (89.2)		51 (2.6)		1906 (97.4)		161 (8.2)		1796 (91.8)		170 (8.7)		1787 (91.3)	
**Regularity of daily life**															
	Regular	1301 (57.3)		19 (1.5)		1282 (98.5)		76 (5.8)		1225 (94.2)		79(6.1)		1222 (93.9)	
	Irregular	969 (42.7)		67 (6.9)		902 (93.1)		161 (16,6)		808 (83.4)		172 (17.8)		797 (82.2)	
**Exercise**															
	No exercise	809 (35.6)		47 (5.8)		762 (94.2)		135 (16.7)		674 (83.3)		143 (17.7)		666 (82.3)	
	Continued exercise	1461 (64.4)		39 (2.7)		1422 (97.3)		102 (7.0)		1359 (93.0)		108 (7.4)		1353 (92.6)	
**Concern about COVID-19**															
	Not very concerned (<1 hour per day)	1036 (45.6)		32 (3.1)		1004 (96.9)		131 (12.6)		905 (87.4)		135 (13.0)		901 (87.0)	
	Very concerned (>1 hour per day)	1234 (54.4)		54 (4.4)		1180 (95.6)		106 (8.6)		1128 (91.4)		116 (9.4)		1118 (90.6)	
**Student type**															
	Medical student	563 (24.8)		20 (3.6)		543 (96.4)		57 (10.1)		506 (89.9)		60 (10.7)		503 (89.3)	
	Nonmedical student	1707 (75.2)		66 (3.9)		1641 (96.1)		180 (10.5)		1527 (89.5)		191 (11.2)		1516 (88.8)	
Total			2270 (100)		86 (3.8)		2184 (96.2)		237 (10.4)		2033 (89.6)		251 (11.1)		2019 (88.9)

^a^Percentages in the Total column are calculated based on N=2270; all other percentages are calculated based on the values in the Total column.

**Table 2:** Mann-Whitney *U* tests and z scores of factors affecting students’ mental health issues as the grouping variable.

Variable (factor)	Mann-Whitney *U*	Mann-Whitney *z*	*P* value
Gender (X8)	243138.00	–1.241	.22
Age (X1)	241098.00	–2.015	.04
Only child (X11)	249085.00	–0.508	.61
Ethnicity (X13)	252327.50	–0.176	.86
Place of residence (X14)	252570.50	–0.097	.92
Region (X10)	252391.50	–0.550	.58
Joining in volunteer work (X9)	249072.50	–0.818	.41
Contact history of similar infectious disease (X2)	247191.00	–3.646	<.001
Past medical history (X3)	233145.00	–5.787	<.001
Compulsive behaviors (X4)	200731.00	–9.003	<.001
Regularity of daily life (X5)	179774.00	–8.774	<.001
Exercise (X6)	192609.00	–7.481	<.001
Concern about COVID-19 (X7)	230177.50	–2.747	.006
Student type (X12)	250828.00	–0.349	.73

The above 7 factors (age, contact history of similar infectious disease, past medical history, compulsory behaviors, regularity of daily life, exercise, and concern about COVID-19) that had a significant association (*P*<.05) in the Mann-Whitney *U* test were entered into binary logistic regression model 1 as independent variables for mental health issues. Likewise, all the other above factors were entered into the binary logistic regression model in order of *P* value from small to large (the order is indicated in Table 2). First, we analyzed the overall effectiveness of these models (Table 3). The original hypothesis of the model test was that the quality of the model would be the same if the independent variable was or was not included. The results showed that the *P* value is <.05, which means that the original hypothesis is rejected, and the construction of these models is meaningful; moreover, based on the Akaike information criterion (AIC) goodness-of-fit statistic for comparing models, model 1 (AIC=1397.629), with the lowest AIC statistic, is the model with the best fit.

**Table 3:** Model summary of binary logistic regression analysis of students’ mental health issues.

Model	–2 log likelihood	Chi-square (*df*)	*P* value	AIC^a^	BIC^b^
Intercept-only	1578.608	N/A^c^	N/A	N/A	N/A
Model 1 (X1, X2, X3, X4, X5, X6, X7)	1381.629	196.979 (7)	<.001	1397.629	1443.449
Model 2 (X1 ,X2, X3, X4, X5, X6, X7, X8)	1380.594	198.014 (8)	<.001	1398.594	1450.142
Model 3 (X1, X2, X3, X4, X5, X6, X7, X8, X9)	1378.846	199.762 (9)	<.001	1398.846	1456.121
Model 4 (X1, X2, X3, X4, X5, X6, X7, X8, X9, X10)	1378.483	200.125 (10)	<.001	1400.483	1463.486
Model 5 (X1, X2, X3, X4, X5, X6, X7, X8, X9, X10, X11)	1378.313	200.295 (11)	<.001	1402.313	1471.044
Model 6 (X1, X2, X3, X4, X5, X6, X7, X8, X9, X10, X11, X12)	1377.973	200.635 (12)	<.001	1403.973	1478.431
Model 7 (X1, X2, X3, X4, X5, X6, X7, X8, X9, X10, X11, X12, X13)	1377.778	200.830 (13)	<.001	1405.778	1485.963
Model 8 (X1, X2, X3, X4, X5, X6, X7, X8, X9, X10, X11, X12, X13, X14)	1377.540	201.068 (14)	<.001	1407.540	1493.453

^a^AIC: Akaike information criterion.

^b^BIC: Bayesian information criterion.

^c^N/A: not applicable.

As shown in Table 4, the above 7 factors in the optimal model (Model 1) were used as independent variables, and mental health issues were used as the dependent variable for binary logistic regression analysis; the formula of the model was 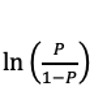 = 0.618 – 0.075 × X1 + 1.213 × X2 + 1.188 × X3 + 1.260 × X4 – 0.892 × X5 – 0.786 × X6 – 0.450 × X7 (where *P* represents the probability that mental health issues are positive and 1 – *P* represents the probability that mental health issues are negative). The analysis indicates that age (odds ratio [OR] 0.928, *P*=.02), regular daily life during the epidemic outbreak (OR=0.410, *P*<.001), exercise during the epidemic outbreak (OR 0.456, *P*<.001), and concern about COVID-19 (OR 0.638, *P*=.002) were protective factors for mental health issues, as shown in Table 3. The results showed that older students and students who maintained regular daily life activity and physical exercise during the COVID-19 pandemic were less likely to have mental health issues. However, contact history of similar infectious disease (OR 3.363, *P*=.02), past medical history (OR 3.282, *P*<.001), and compulsive behaviors (OR 3.525, *P*<.001) were risk factors for mental health issues; this finding showed that students with these three conditions were more likely to have mental health issues.

**Table 4:** Estimations of binary logistic regression analysis of students’ mental health issues as the dependent variable.

Variable	Mental health issues				
	B	SE	*z*	*P* value	Exp(B) (95% CI)
Age	–0.075	0.033	–2.301	.02	0.928 (0.870-0.989)
Contact history of similar infectious disease (yes vs no)	1.213	0.501	2.422	.02	3.363 (1.260-8.976)
Past medical history (yes vs no)	1.188	0.251	4.736	<.001	3.282 (2.007-5.367)
Compulsive behaviors (yes vs no)	1.260	0.167	7.554	<.001	3.525 (2.542-4.887)
Regularity of daily life (regular vs irregular)	–0.892	0.150	–5.936	<.001	0.410 (0.305-0.550)
Exercise (yes vs no)	–0.786	0.144	–5.446	<.001	0.456 (0.344-0.605)
Concern about COVID-19 (yes vs no)	–0.450	0.147	–3.066	.002	0.638 (0.478-0.850)
Constant	0.618	0.691	0.894	.37	1.855 (0.479-7.182)

## Discussion

### Principal Findings

In our study, we analyzed the psychological responses and associated factors, including risk factors and protective factors, of both medical and nonmedical students after the outbreak of COVID-19. We found that past medical history, contact history of similar infectious diseases, and compulsive behaviors contributed to the risk of mental health issues. Maintenance of regular daily life and exercise during the epidemic outbreak, older age, and high levels of concern (>1 hour per day) about COVID-19 were protective factors for mental health issues.

The COVID-19 outbreak is the largest outbreak of atypical pneumonia since SARS in 2003 in terms of the number of infection cases and time of spread [30]. COVID-19, compared with SARS, has brought greater risk of death and very high psychological pressure to people worldwide due to its power of “superspreading” among humans [6,31]. Studies have demonstrated the psychological impact of the early stage of COVID-19 on the general population, including college students [32-34], and it has been indicated that both medically trained medical staff and nonmedical health care personnel can be affected [35,36]. Our study provided evidence that 251/2270 students (11.1%) encountered mental health issues during the outbreak of COVID-19. During the continuous spread of the epidemic, strict isolation measures and closures in campuses across China may have affected university students’ mental health [37,38]. More importantly, medical students who are medical workers with incomplete training possess only basic medical knowledge and do not have proficient professional skills and abundant clinical experience. Therefore, their clinical exposure risks are higher than those of other people. Likewise, the mental health of medical students requires attention during the pandemic.

A few studies reporting the psychological conditions of college students or medical students during the COVID-19 outbreak emerged during the preparation of our manuscript. Copeland et al [39] investigated the impact of the COVID-19 pandemic on the emotions, behaviors, and wellness behaviors of first-year college students and showed that COVID-19 and related mitigation strategies have a moderate but continuous impact on mood and healthy behavior. Bolatov et al [40] compared the mental state of medical students switching to web-based learning with that of students who received traditional learning during the COVID-19 pandemic, and they revealed that the prevalence of burnout syndrome, depression, anxiety, and somatic symptoms decreased after the transition. Li et al [41] investigated the rates of three mental health problems (acute stress, anxiety, and depressive symptoms) and their change patterns in two phases of the pandemic (early vs under control), and they showed that the significant predictors of distinct mental health trajectories included senior students, COVID-19 exposure, COVID-19–related worries, social support, and family function. Isralowitz et al [42] examined COVID-19–related fear and its association with psychoemotional conditions, including use of substances such as tobacco, alcohol, and cannabis, among Israeli and Russian social work students at two peak points or waves of infection. These literature reports focused on a specific population or condition, a relatively small sample size, and limited survey items during the COVID-19 pandemic. Compared with the above studies, our survey covered a large population of both medical and nonmedical university students, from undergraduates to graduates, with a wide geographical range using multidimensional survey items, including detailed demographic information and mental and behavioral status, and we analyzed the factors associated with the psychological responses. Our results are more specific and detailed.

Our study focused on the psychological status of university students and concluded that university students with compulsive behaviors are more likely to have mental health issues, which confirmed the conclusions of previous research. The research has suggested that youth with OCD are at risk of experiencing comorbid psychiatric conditions, such as depression and anxiety [43]. Students with past medical history are more likely to have anxiety and depression symptoms; this is consistent with recent research findings, in which a medical history of issues such as prior psychiatric illness, physical illness, or chronic disease was a risk factor for the deterioration of mental health [13-15], and indicates that these students are more sensitive to the epidemic and require more psychological intervention.

University students with irregular daily life during the epidemic outbreak were more likely to have mental health issues, which is similar to previous research results. Previous studies have suggested that with the increasing pressure of modern life and irregular lifestyles, depression has become an increasing threat to human health. Studies have also suggested that better mental health at baseline was predicted by a lower body mass index, a higher frequency of physical and mental activities, nonsmoking, a nonvegetarian diet, and a more regular social rhythm [44]. Students should maintain a regular and healthy lifestyle during the epidemic to ensure good mental health status.

Our study also indicates that students with a contact history of similar infectious diseases are more likely to have mental health issues because this experience may cause students to worry about whether they have been infected, and medical students are more likely to be exposed to similar diseases in clinical work. Previous studies also suggested a significant association with anxiety for people whose contact histories included contact with an individual with suspected COVID-19 or with infected materials [31].

This study indicates that students who exercised during the epidemic outbreak were less likely to have mental health issues. Studies have suggested that physical exercise is associated with greater cardiovascular fitness, improved muscle strength and endurance, and reduction of depression and anxiety [45]. Students should continue to exercise during the epidemic to ensure good mental health. Unexpectedly, a high level of concern about COVID-19 was less likely to be associated with mental health issues owing to the dissemination of positive scientific information on Chinese media’s public emergencies. Therefore, it is recommended to pay suitable attention to the news, especially the good news related to the epidemic, and such behaviors are beneficial to maintain a good attitude during the outbreak of COVID-19.

Previous studies have suggested that female and older students are more likely to have mental health symptoms [11,12]; women and older people have been found to experience more significant psychological impact and higher stress levels, anxiety, and depression [31]. These findings are inconsistent with our research results; because older students experienced SARS in 2003, they may have a more comprehensive understanding and a higher level of awareness of COVID-19 and may be less likely to have mental symptoms during the epidemic.

For family and society, these risk variables that cause mental health issues are key factors for early judgment of university students’ psychological problems, and the results also provide the theoretical basis for formulating intervention measures. Schools, families, and the government should provide more care and support to university students during the epidemic.

### Limitations

Given the limited available resources and the time of the COVID-19 outbreak, the study adopted the snowball sampling strategy, which is not based on randomly selected samples. Additionally, the researchers did not conduct a prospective study that would provide a specific measure to support the needs of targeted public health initiatives.

### Conclusions

During the outbreak of COVID-19, some university students experienced mental symptoms. Past medical history, contact history of similar infectious disease, and compulsive behaviors were risk factors for mental health issues. Older age of the students, regular daily life, and exercise during the epidemic outbreak were protective factors against mental health issues. A high level of concern (>1 hour per day) about COVID-19 was also a protective factor.

These findings are beneficial for identifying the groups of university students at risk for possible mental health issues, and they provide a theoretical foundation for the formulation of relevant interventions so that universities and families can prevent or intervene in the development of mental health issues among students at the early stage of the disease. Likewise, the findings are essential for education and public health epidemic prevention. In short, students require more attention, help, and support from society, families, and universities during the COVID-19 pandemic.

### Availability of Data and Materials

The data and materials used in this study are available upon request from the author.

## Abbreviations

AIC: Akaike information criterion
OCD: obsessive-compulsive disorder
OR: odds ratio
SARS: severe acute respiratory syndrome
SAS: Self-Rating Anxiety Scale
SDS: Self-Rating Depression Scale
YBOCS: Yale-Brown Obsessive-Compulsive Scale
